# Sensing What You Do Not See: Alerting of Approaching Objects with a Haptic Vest

**DOI:** 10.3390/s25185808

**Published:** 2025-09-17

**Authors:** Albina Rurenko, Devbrat Anuragi, Ahmed Farooq, Marja Salmimaa, Zoran Radivojevic, Sanna Kumpulainen, Roope Raisamo

**Affiliations:** 1 Faculty of Information Technology and Communication Sciences, City Centre Campus, Tampere University, 33100 Tampere, Finland; albina.rurenko@tuni.fi (A.R.); devbrat.anuragi@tuni.fi (D.A.); ahmed.farooq@tuni.fi (A.F.); sanna.kumpulainen@tuni.fi (S.K.); 2 Nokia Technologies, 33100 Tampere, Finland; marja.salmimaa@nokia.com; 3 Nokia Bell Labs, Cambridge CB3 0FA, UK

**Keywords:** wearable haptics, haptic alerts, human-technology interaction

## Abstract

**Highlights:**

**What are the main findings?**

- Perceived urgency was negatively correlated with burst duration (BD)—shorter vibrations were consistently rated as more urgent, identifying BD as a key parameter for urgency encoding.
- Location-based haptic alerts significantly outperformed pattern-based alerts in terms of hazard detection rate and reaction time.

**What is the implication of the main finding?**

- Performance associate with location-based vibrations suggests a good potential of such signals in safety-critical alerting wearable systems.
- Pattern-based signals potentially require more cognitive resources to interpret, which may hinder their use in high-load or multitasking environments.

**Abstract:**

Workplace accidents in high-risk environments remain a major safety concern, particularly when workers’ visual and auditory channels are overloaded. Haptic feedback offers a promising alternative for alerting individuals to unseen dangers and enhancing situational awareness. Motivated by challenges commonly observed in construction, this study investigates haptic alerting strategies applicable across dynamic, attentionally demanding contexts. We present two empirical experiments exploring how wearable vibration cues can inform users about approaching objects outside their field of view. The first experiment evaluated variations of pattern-based vibrations to simulate motion and examined the relationship between signal parameters and perceived urgency. A negative correlation between urgency and pulse duration emerged, identifying a key design factor. The second experiment conducted a novel comparison of pattern-based and location-based haptic alerts in a complex virtual environment, with tasks designed to simulate cognitive engagement with work processes. Results indicate that location-based alerts were more efficient for hazard detection. These findings offer insights into the design of effective user-centred haptic-based safety systems and provide a foundation for future development and deployment in real-world settings. This work contributes a generalisable step toward wearable alerting technologies for safety-critical occupations, including but not limited to construction.

## 1. Introduction

The term *situation awareness* refers to understanding and anticipating the state of the environment and its elements over time [1]. It is critical for safety in dynamic environments, where multiple agents, machinery, and evolving conditions coexist. One such setting is a construction site [2], where workers often operate under high mental load and stress [3,4], factors known to affect attention and working memory [5,6]. These cognitive constraints increase vulnerability to hazards, particularly when primary sensory channels are already overloaded. Enhancing safety in such contexts therefore would benefit from dynamic alerting systems that timely identify and communicate potential hazards, balancing situation awareness.

While many research efforts have addressed hazard detection technologies (e.g., [7,8,9,10,11,12,13]), relatively little attention has been paid to how alerts are delivered. Yet, effective delivery is crucial. According to the Communication-Human Information Processing model [14,15], received warnings must pass through several cognitive stages before being converted into behaviour. Any of the stages can become a bottleneck preventing the receiver from taking safety reinforcement actions. Thus, an inappropriate warning delivery channel may fail to capture attention or sustain it long enough to prevent warnings from being filtered out and ignored.

Visual and auditory channels, the predominant modalities for safety alerts in domains like construction, are often compromised in noisy or visually complex environments [16,17]. Haptic feedback offers a compelling alternative, possessing several key advantages [18,19,20,21]. By bypassing the overloaded visual and auditory channels, haptic signals convey information directly through tactile sensations [22]. Tactile cues have been shown to trigger faster attention shifts and reactions than visual or auditory stimuli due to direct neural pathways [23], which is critical in time-sensitive safety scenarios.

However, effectiveness of tactile cues depends on their design. Sabnis et al. [24], drawing on Ihde’s taxonomy [25], classify haptic signals by mediation type. *Embodied* mediation enables prereflective perception where signals are immediately experienced as meaningful, and *hermeneutic* mediation requires conscious interpretation. This suggests that haptic signals employing embodied mediation, capable of enabling quick reactions, could be especially effective in addressing one of the leading causes of work accidents—injuries resulting from collisions with moving objects [26].

In this study, we compare two distinct approaches to wearable haptic alerting technology for high-risk worksite safety: **pattern-based** vibrations hypothesised to leverage embodied mediation and **location-based** vibrations representing hermeneutic mediation. Embodied mediation signals represent haptic patterns creating an apparent tactile motion. The opposed hermeneutic mediation signals lean on a correspondence between the stimulated body location and direction of the object’s movement, potentially requiring more cognitive processing. This approach raises the following research questions:

- RQ1: Which type of haptic feedback is more effective—pattern-based or location-based?
- RQ2: Which type of haptic feedback is more efficient—pattern-based or location-based?
- RQ3: Does varying signal duration influence reaction times?

By answering these research questions, we will contribute to alert design strategies for hazardous work environments and empirical proof whether haptic alerts of hermeneutic mediation type lose due to their interpretation.

We acknowledge the importance of a multimodal approach to cover as many scenarios when the only modality may fail [27,28,29]; however, this research focusses on haptics as a potent part of a possible cross-modal alerting system.

The paper is structured as follows. Section 2 reviews related work. Section 3 and Section 4 describe the experiment for developing pattern-based haptic signals. The methodology and results of the comparison experiment are presented in Section 5 and Section 6, followed by a discussion of our findings, limitations, and future directions in Section 7. Conclusions are summarised in Section 8.

## 2. Related Work

Wearable safety systems can benefit various high-risk environments, such as manufacturing, logistics, and emergency response, but in this paper, we primarily draw on construction literature as a representative use case with an undeniable need to improve the safety measures related to collisions [26] to ground our design rationale.

### 2.1. Alerts in High-Risk Work Environments

Safety-critical domains such as mining [11,12,30], firefighting [13,31], and manufacturing [32,33] typically rely on visual and auditory modalities to communicate warnings and alerts. In construction, visual cues, such as pictorial or textual signs, are a common approach. For example, Kim et al. [8] proposed a visual monitoring system for object tracking that displays safety levels. Wu et al. [9] presented a similar model, albeit based on coordinates mapped to visual monitoring. Chan et al. [34] introduced a visual alerting system based on the field of view, which notifies about proximate hazards and adapts to the worker’s attention. Indeed, it is a resource for which visual alerts compete. In cluttered scenes, cognitive overload can lead to “inattentional blindness” [35] when individuals fail to notice new visual information.

Although some designs of visual warnings prove more effective than others [36], researchers underscored an additional challenge: the role of cultural differences in risk perception [37,38]. They emphasise the importance of using uniform alert systems that perform consistently across a diverse workforce, ensuring that team members from different cultural backgrounds similarly interpret the warnings. This limitation highlights the inherent drawback of relying on visual modalities for construction site warnings.

Visual cues are often complemented with audio, as in a study by Edirisinghe & Blismas [39], who developed a prototype of a vest that responds to temperature changes, alerting users through LED lights and music or loud “beeps” (see [16] for a comprehensive review of recent wearable systems). However, construction sites are inherently filled with a multitude of sounds, making it difficult to isolate important auditory signals [17]. Moreover, the use of hearing protection further diminishes auditory alert effectiveness, leaving space for warning improvements.

### 2.2. Haptics for Communicating Spatial Information

Currently, hermeneutic mediation appears to dominate the use of haptic cues in the construction field. Haptics has been applied to provide workers with real-time awareness of their surroundings: for example, Cassinelli et al. [40] developed a system that augmented spatial awareness through motor actuators placed on different parts of the body. Using simple on/off vibrations, the system communicated the presence of obstacles along the user’s path through proximity sensors, enhancing the worker’s ability to avoid unseen hazards. Similarly, Huang et al. [41] designed a wristband that used varying intensities of haptic feedback to indicate the proximity of heavy machinery, offering this understanding through three distinct intensity levels. Ogrinc et al. [42] extended this approach by additional conveying of nearby moving objects’ speed.

Yang & Roofigari-Esfahan [43] tested vibrational alerts along with visual and auditory in a virtual road construction setting. Vibration alerts provided with information on the hazard direction or recommended safe direction, or both. Body locations such as shoulders and waist were used to deliver the information.

Cho & Park [44] trained participants to respond to haptic alerts by dodging to avoid an approaching object representing a construction site hazard. Using a vest equipped with four vibratory motors, they designed three distinct haptic cues based on “on/off” vibration patterns. After training, participants demonstrated high accuracy and rapid reaction times. Haptic technology has also been explored as a means of communicating distance. Kim et al. [45] developed a white cane equipped with four actuators in the handle, which conveyed information about object distance through variations in vibration spatial location, timing, and intensity. Their system achieved high recognition rates for these spatial cues. Building on this concept, Ohara et al. [46] proposed “stereohaptic vibration,” a technique that uses multiple distributed actuators to polarise the perceived intensity of vibrations, enabling users to localise vibration sources outside the body. This concept, tested with four actuators on the hand and one on the palm, showed promising results in conveying directional and distance-based spatial information. See Lederman & Jones [47] for additional discussion on haptic effects and illusions.

Moreover, haptics have been widely applied to assist with navigation and directions in various environments. Kaul et al. [48] used head-mounted actuators to guide users in a 3D virtual environment, where haptic feedback outperformed auditory cues for directional information. Farooq et al. [49] placed actuators on shoulder blades to deliver directional cues through vibrations on the corresponding side. Rantala et al. [50] presented an method to direct the user’s gaze with spatial haptic information presented on smart glasses.

These studies illustrate how haptic feedback can effectively communicate spatial information in an unobtrusive way, making it a powerful tool to improve situational awareness in complex high-risk environments. However, challenges remain. Haptic cues can easily become ambiguous when multiple actuators are used simultaneously, and users may struggle to reliably map vibration patterns to spatial meaning under time pressure or cognitive load. Therefore, it is important to investigate how different haptic signals perform under varying task demands and environmental conditions, particularly when rapid and accurate responses are critical.

## 3. Experiment  1

### 3.1. Objective

**Figure 1:**
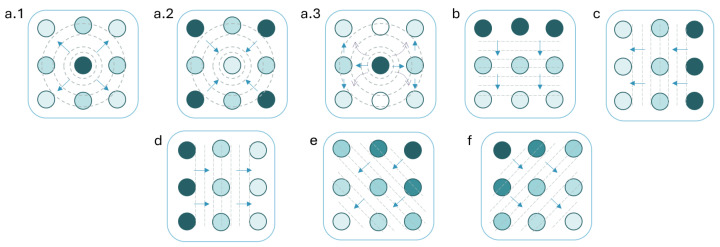
Motion direction patterns. Colours and arrows demonstrate activation order of actuator groups. (**a.1–a.3**)—options for straight motion towards one’s back; (**b**)—top-down; (**c**)—right-left; (**d**)—left-right; (**e**)—upper right-lower left; (**f**)—upper left-lower right.

To support this comparison, we designed haptic patterns intended to simulate the motion of an approaching object—our hypothesised form of embodied mediation. As shown in Figure 1, the patterns represent several directions: directly from behind, laterally from the left and right, diagonally from the top-left and top-right, and vertically from above. Patterns ***b–d*** and the reversed forms of ***e–f*** adapt designs validated by Jones et al. [51] on the forearm. In contrast, patterns ***a.1–a.3*** were newly developed to simulate direct linear motion toward the back. Since no established pattern for this motion existed in a comparable setup, we first conducted an empirical test of all three pattern variations. Then, we also explored modulation of signal perceived urgency to enable future comparison with location-based haptic alerts across different urgency levels.

### 3.2. System Description

**Figure 2:**
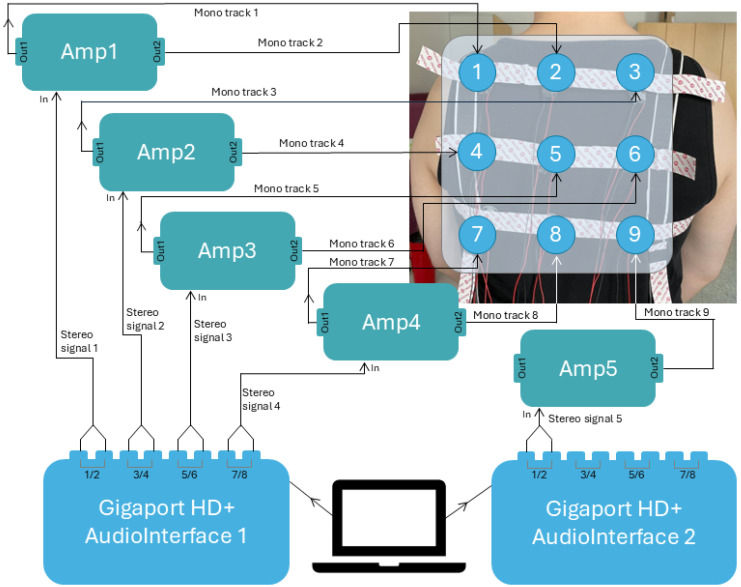
System diagram. Audio interfaces—Gigaport HD+ (ESI Audiotechnik GmbH, Leonberg, Germany), acoustic amplifier modules (Amp1–5)—Hailege TDA7297 AC/DC 12V 2*15W model (Shenzhen Hailege Technology Co., Ltd., Shenzhen, China), actuators—Tectonic voice coils TEAX13C02-8/RH model (Tectonic Audio Labs, Woodinville, WA, USA).

For this study, we developed a 3 × 3 matrix of haptic actuator feedback system. We chose to place the feedback system on the participants’ upper back to inform them of a potentially dangerous motion out of the sight. The actuators were attached to a Velcro-made vest, allowing adjustments of the location and fit according to every participant’s constitution. Each actuator was connected to one of the five acoustic amplifiers which were mains powered (connected to a breadboard). Each amplifier received two mono signals through audio interfaces connected to a laptop with a running Python (version 3.6) code. Refer to Figure 2 for details of the setup.

### 3.3. Patterns and Signals

Design of all pattern-based haptic signals was based on two perceptual phenomena: the *apparent tactile motion effect* [52,53] and the *funnelling illusion* [47]. Prior work, including the “Snake Effect” [54] and “Tactile Brush” [55] studies, explored how variations in signal timing can evoke the illusion of motion across the skin. In our work, we rely on the *burst duration* (BD) and *inter-burst interval* (IBI) values (see [56] for terminology) derived by Severgnini et al. [54] as baseline (BD = 1.69 s, IBI = 500 ms) for all patterns. Israr & Poupyrev’s approach [55] was tailored to short linear strokes and, therefore, is less applicable to simulate motion directed to the back.

Each pattern was represented by a sequence of actuator group activations mimicking physical movement across receptors. Actuators within the same group (identically shaded in Figure 1) were activated simultaneously, where activation refers to delivering a single vibrational pulse. All actuators in a group received an identical signal. IBI defined the interval between the onsets of consecutive group activations. Following Severgnini et al. [54], each pulse was constructed as an amplitude-modulated sine wave with an envelope function $E\left(t\right)=A\sin\left(2\pi F_{e}t\right)$, where *A* is the amplitude and $F_{e}$ is the envelope modulation frequency.

Direct approaching to the back intuitively should “draw” motion to the back’s centre; thus, for pattern variations *a.1–a.3*, we chose to examine varying BD and IBI across actuator groups:

- BD1—burst duration of group 1 (1.00/1.30/1.69 s)
- BD2—burst duration of group 2 (1.00/1.30/1.69 s)
- BD3—burst duration of group 3 (1.00/1.30/1.69 s)
- IBI1—inter-burst interval between group 1 & 2 (250/500 ms)
- IBI2—inter-burst interval between group 2 & 3 (250/500/750 ms)

We chose the BD values to be smaller or equal to Severgnini’s et al. [54] value considering necessity of quick information delivery. The smallest BD value is 1 s, twice as large as the 500 ms baseline IBI.

Combining the features results in 162 variations. However, we discarded combinations where the first inter-burst interval (IBI1) exceeded the second (IBI2) or the third burst duration (BD3) exceeded the second (BD2), based on empirical tests conducted by two authors—both conditions produced confusing and undefined motion sensations. This reduced the number of evaluated combinations to 90. For efficiency, two authors of the paper performed a preliminary screening and rated feature combinations for each pattern variation according to how strongly it conveyed motion toward the back. The scale was subjective, from 1 to 5 (least to most like a motion toward the back). To ensure a conservative exclusion, a combination was excluded only if both authors rated it 1 or 2. Thus, after screening, variation ***a.1*** had 25 combinations, ***a.2*** had 18, and ***a.3*** had 8.

### 3.4. Participants

For this study, we recruited 21 participants in Pirkanmaa region of Finland. In total, 11 persons’ identified gender was male, 8—females, 1—non-binary, and 1 preferred not to say. The age ranged from 23 to 41 years with a median of 29. All participants had at least a bachelor’s degree and were mostly master’s students.

### 3.5. Procedure

#### 3.5.1. Testing Pattern Variations a.1–a.3

Actuator amplification was adjusted through acoustic amplifiers with a physical knob when necessary for each participant. Relying on participant’s feedback, we ensured equally strong vibration sensation, accounting for differences in location-based skin sensitivity [57]. The participants then selected the most comfortable signal frequency (200, 250, or 300 Hz) within the optimal tactile range [58,59], separately for each pattern variation, which was used for all corresponding signals.

Each participant was first introduced to two random (Here and in further mentions: Randomisation used Python’s *random* library based on the MT19937 Mersenne Twister algorithm) signals of each pattern variation in random order for familiarisation. Signal evaluation was conducted in several steps. First, participants rated all feature combinations for each pattern variation based on how strongly it conveyed motion sensation toward the back (1–5), similar to the screening authors. Combinations and pattern variation orders were randomised to balance the first exposures. Participants were encouraged to take breaks to avoid fatigue and sensitivity decline of fast-adapting mechanoreceptors [60].

**Figure 3:**
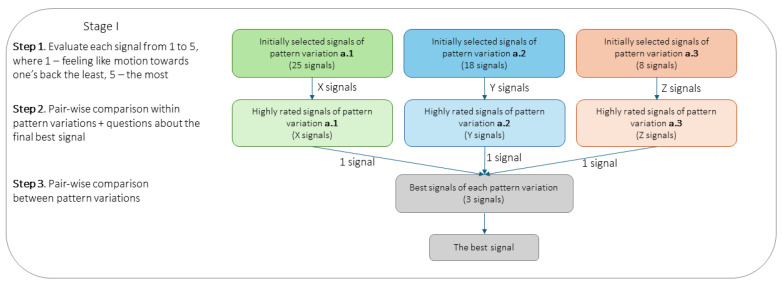
Flowchart of testing pattern variations a.1–a.3.

Next, participants compared pairs of the top-rated signals within each variation, choosing the most effective combination for each. Finally, these three winning combinations were compared pairwise to identify the overall best pattern and feature combination. Flowchart of the procedure is shown in Figure 3.

#### 3.5.2. Perception of Signal Urgency

For the examination of changes in signal urgency perception, we chose to control signal frequency and BD in conjunction with IBI. Signal duration and interpulse rate, relative to IBI, have previously been shown to influence the urgency perception of pulse vibrations [61], although not linearly, while frequency is a proven hazard perception factor in visual and auditory alerts [62,63,64]. We introduced motion represented by patterns ***b–f*** in a range of different speeds, with zero acceleration. BD and IBI were the same for all actuator groups for each pattern.

**Figure 4:**
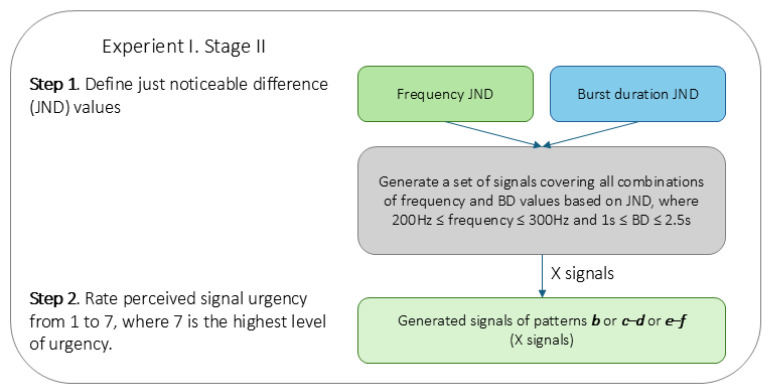
Flowchart of testing urgency perception.

To create personalised datasets of signal features with varying frequencies and BD, we applied a *just noticeable difference* (JND) method—also known as the “Method of Limits” [65,66]—which applies an adaptive step-size approach to identify the smallest noticeable changes. Flowchart of the procedure is shown in Figure 4.

To determine each participant’s frequency JND, we started with a base of **200 Hz** and an initial **5 Hz** increment. If a difference was detected (e.g., between 200 and 205 Hz), the step was reduced by **1 Hz** until the smallest detectable change was identified. If no difference was detected, the step was increased by **5 Hz** until it was, and then it was reduced stepwise to pinpoint the JND. Thus, JND was defined as the smallest frequency increment at which the participant could reliably and consistently detect a difference through this iterative stepwise procedure.

For the burst duration JND, we applied the same method, with an initial increment of **10%** of the baseline BD of 1.69 s. JND was measured in percentages of baseline BD and could reach as little as **2%** of the baseline (33.8 ms) for highly sensitive participants.

Personalised sets of signals constituted various combinations of frequencies and BD values. Frequency ranged from **200** to **300 Hz**, stepped by frequency JND, and the BD ranged from **1** to **2.5 s** stepped by BD JND (in %), centred on the 1.69 s baseline, so that the value appeared in each set. The respective IBI values were calculated similarly, ensuring proportional changes to the BD adjustments, with a starting baseline value of **500 ms**.

Duration of each group activation was constant and equal to BD. Each participant was assigned to one of the directions—from above (pattern ***b***), sideways (pattern ***c*** or ***d***), or diagonal (pattern ***e*** or ***f***).

Before testing, a participant experienced 10 random signals of their personal set for familiarisation. A subjective scale from 1 (‘not urgent at all’) to 7 (‘very urgent’) was used to rate perceived urgency. The 7-point scale was chosen due to its reliability in subjective evaluations [67] and suitability for analysis, which defined the BD values for the experiment. Additionally, participants were asked if each signal felt like a continuous movement across the skin to exclude feature sets that did not convey this sensation.

## 4. Results of Experiment 1

### 4.1. Pattern Variations a.1–a.3

During the final pair-wise comparison, ***a.2*** was preferred most by a small margin 42.8% (9/21 participants). One less participant (38%) preferred ***a.1*** pattern variation. A Chi-square goodness-of-fit test, comparing the distribution across all three types did not show a significant deviation from the equal preference ($\chi^{2}$ = 2.0, *p* = 0.4). These results suggest that while ***a.2*** had the highest preference proportionally, the differences were not significant. Pattern ***a.2*** was selected as the representative pattern for the second experiment due to its slightly higher preference rate. In relation to signal features within pattern variations, there were no clear tendencies. The only discovered commonality concerning ***a.2*** pattern was a preference of 500 ms for IBI1 and IBI2.

### 4.2. Perceived Urgency

The collected data included signal features, urgency scores, and responses to whether the signal felt like a motion. Signals that did not induce a sense of motion were excluded. Pearson’s correlation for urgency scores and BD values was calculated for the remaining signals within each participant’s dataset, as well as for urgency scores and frequency values. In all datasets except one, regardless of the pattern, there was a negative correlation between urgency scores and BD. The correlation scores within the tested patterns fluctuated as follows: pattern ***b*** −0.91<=r<=−0.60, p<0.01; patterns ***c–d***−0.87<=r<=−0.33, p<0.01; patterns ***e–f***−0.95<=r<=−0.66, p<0.01 with one participant’s dataset not yielding any statistically significant correlation.

On the other hand, frequency did not exhibit a linear relationship with perceived urgency scores. Out of 21 participants’ datasets, only 3 showed significant statistic results (r<−0.2 | r>0.2, p<0.01), representing two different patterns. Two results showed a positive correlation and one negative. However, these findings are not consistent and do not represent the entire tested population. Examining data across BD values, no consistent correlation between frequency and urgency scores was found either.

To investigate whether there was a difference between signals perceived as motion and those that were not, we performed a permutational multivariate ANOVA test (PERMANOVA), as the data was not normally distributed (Shapiro–Wilk test for urgency scores, p<0.01). According to the results, there was no statistically significant difference between the two groups within the signals of all patterns. When only BD and IBI were used as comparison features, a significant difference was found between the signals in patterns ***e–f***. Full statistical details are provided in Table 1.

## 5. Experiment 2

### 5.1. Objective

To compare the two alert types, we measured reaction times and detection times as indicators of efficiency, and detection rate as an indicator of effectiveness. These measures were compared between two groups: one receiving *pattern-based vibration* (PBV) alerts and the other—*location-based vibration* (LBV) alerts. These comparisons helped assess the relative effectiveness of each signal type in terms of speed and accuracy.

### 5.2. Participants

We recruited 20 participants from Pirkanmaa region of Finland and divided them into two equal groups: PBV and LBV. To mitigate familiarity effects, no participants from Experiment 1 were included in the PBV group. Of the 20 participants, 14 identified as male and 6 as female. The age ranged from 22 to 63 years, with a median age of 28.

All participants had at least a bachelor’s degree, the majority (n = 14) were master’s students. Familiarity with virtual reality (VR) varied widely between participants, with majority of the participants (n = 13) reported having experienced VR fewer than 10 times.

### 5.3. Procedure

#### 5.3.1. Setup

The experiment utilised a 360° field-of-view virtual environment simulating a construction site. To enhance immersion and mask vibration noise, an ambient soundscape was introduced, featuring typical construction sounds and noises.

To simulate various cases of workers’ mental occupation, we designed three types of tasks requiring different cognitive resources. The tasks were identical for all participants, only the order was diversified. The instructions and tasks were displayed on a virtual canvas positioned directly in front of the participant.

During each task, an object (e.g., rock or barrier) appeared randomly in various positions around the participant and moved toward them. Simultaneously, the corresponding haptic alert was delivered via the vest. Participants were instructed to locate the object in response to the alert, which would have been natural behaviour in a real-life scenario when warned about an approaching danger. The object disappeared once spotted or upon collision with the participant’s virtual body. Object behaviour and gaze tracking were handled in Unity using the VR headset tracking system.

**Figure 5:**
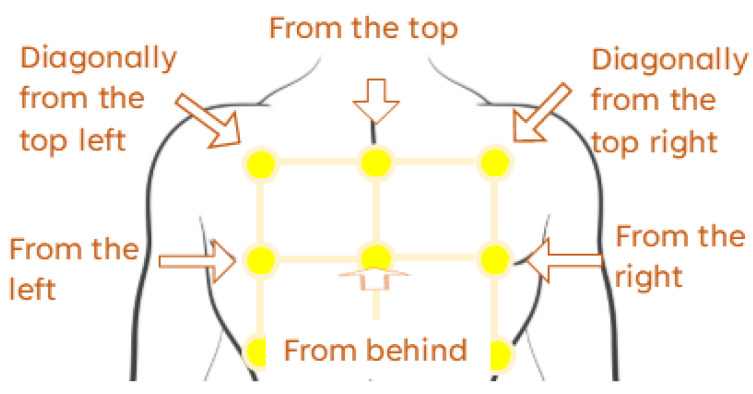
Correspondence between vibrating actuators and motion directions.

The PBV group was informed that the haptic signal indicated a direction of motion, there was no preliminary training on stimuli discerning. The LBV group received an illustration of the correspondence between the stimulated back locations and the movement directions during the instruction phase (see Figure 5). This difference in instructions stems from difference between embodied and hermeneutic mediation types.

Before the experiment, participants in both groups experienced test stimuli and individual actuator signal amplifications were adjusted if needed. For the LBV group, it was an additional training to distinguish each location-based signal. We ensured all actuators were clearly discernible and provided uniform signal intensity.

The participants navigated the scene using a Varjo XR3 VR headset and an HTC VIVE Pro controller. They were instructed to remain seated in a stationary position, turning their heads or spinning in their chairs to interact with the environment.

Participants were given the opportunity to have a break between the tasks. Breaks lasted no longer than 5 min. Participants were also instructed to inform the experimenter about any kind of sickness during the VR experience. The study would have been immediately stopped. No sickness was reported by a single participant.

#### 5.3.2. Tasks

The tasks were designed to simulate scenarios with varying types of cognitive demands:

- **Visual Search Task** imitates situations when worker’s attention is primarily focused on visual processing, such as scanning for specific tools.
- **Mathematical Expression Task** engages higher-level cognitive executive functions, responsible for goal-setting, planning, and effective performance [68] (see [69] for relation of mathematical abilities and executive functions).
- **Memorization and Recall Task** imitates scenarios that place demands on working memory, which under high loads affects safety performance [70].

**Figure 6:**
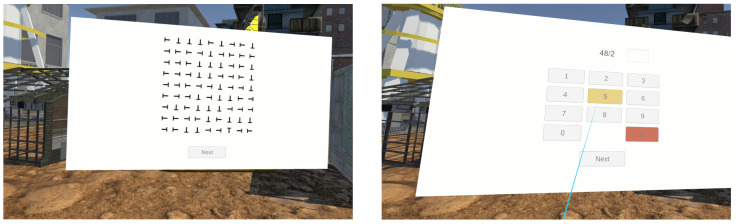
Task 1 Visual search and Task 2 Mathematical expression.

Visual Search Task. This task consisted of 10 subtasks, represented by generated images. Each image contained a 9 × 9 grid of capital “T” letters in different orientations. Only one letter was upright, and participants had to locate it before proceeding. An example can be seen in Figure 6. This task was inspired by Peltier et al. [71] and Hu et al. [72].

Mathematical Expression Task. This task also consisted of 10 subtasks, represented by a randomly generated mathematical expression with two integers (0≤n<100) and one of four basic mathematical operations. The participants solved these problems mentally. All expressions are designed to yield integer solutions.

Digit Sequence Memorisation and Recall Task. This task contained 9 subtasks, each featuring a randomly generated sequence of 5–7 digits that participants had to memorise. The digits were displayed sequentially with a 100 ms delay.

#### 5.3.3. Vibrational Stimuli

The vibration stimuli varied in direction and urgency. The tested directions were similar to Experiment 1 (Figure 1), but the symmetrical directions were combined: left/right as *sideways*, and top-left/top-right as *diagonal*. Urgency was classified as *low*, *medium*, or *high*. All signals in both groups had a modulation frequency of 250 Hz, as it was the most common choice in Experiment 1.

The signal representations for each urgency level were derived from Experiment 1, grouping the signals in low (scores 1–3), medium (4), or high (6–7) perceived urgency levels based on the ratings collected. BD values for each direction and urgency level were analysed across participants for commonalities in signal perception. To determine the most representative BD value for each urgency level, we selected values that appear in the ranges of most of the participants. When multiple BD values fitted the criteria, the smallest BD value was chosen for high urgency, the largest for low, and the closest to average for medium, based on the discovered correlation between BD and perceived urgency scores.

We calculated corresponding IBI values applying a formula BD×500/1.69, making IBI proportional to BD the same way as **500 ms** is proportional to **1.69 s** in Severgnini et al. [54]’s work. BD values ranged from 1.15 to 2.4 s across urgency levels.

Special considerations were made for straight motion towards the back (called *forward* further in the text), urgency levels were defined using data from the first evaluation step of pattern variations of Experiment 1. Since the BD and IBI values vary significantly across actuator groups, signals rated highly (score of 5) were analysed. We chose signals of the shortest, longest, and average durations that were rated by the most participants.

Patterns ***a–d*** were repeated three times per signal, while ***e–f*** were repeated twice. It was a pragmatic design choice to manage the study duration. Since patterns ***e–f*** involved five actuator groups (vs. three in ***a–d***), each cycle was longer, so fewer repetitions helped balance the overall exposure time across patterns.

For the LBV group, each direction was represented by the vibration of a single actuator, as illustrated in Figure 5. The vibration signal consisted of two identical pulses. BD values for each urgency level matched their pattern-based counterparts. Values for inter-pulse interval (IPI)—a pause between two identical pulses—were set equal to the corresponding IBI values of the PBV group. For the forward motion, BD values of 1.15 s, 1.69 s, and 2.23 s were chosen for consistency with other directions.

In total, there were 12 combinations of motion directions and urgency levels. Each participant encountered 36 task instances, ensuring comprehensive exposure to all possible combinations.

#### 5.3.4. Data Collection

Gaze data were recorded throughout the experiment to analyse attention shifts during alert activations. Additionally, head motion data and timestamps of user actions and events (e.g., when a moving object was noticed) were systematically collected. Task performance data was also collected to ensure that participants were engaged with the tasks rather than anticipating haptic stimuli. Finally, semi-structured interviews were conducted after each session to gather participants’ experiences.

*Reaction time* (RT) was defined as the interval between the onset of haptic alert and the participant’s first detectable response—gaze shift, head turn, or body movement. The events were timestamped, and RT was calculated as the difference between them. *Detection time* (DT) was defined as the interval between the onset of the haptic alert and timestamped collision of gaze with an object.

## 6. Results of Experiment 2

The data collected during the experiment was not uniformly distributed across participants for three reasons:

- Some participants, despite instructions, initially ignored the alerts
- Certain participants failed to react to some alerts as they were deeply focused on their ongoing tasks
- The number of reactions and detected objects varied significantly among participants.

Due to missing values and not normal data distribution (Shapiro–Wilk test conducted for DT and RT values in each group, across the tasks and directions, p<0.01), we chose to use the nonparametric Mann–Whitney U test to compare reaction times between the groups. To quantify the magnitude of these between-group differences, we reported Cliff’s delta effect size [73]. Aligned Rank Transform (ART) ANOVA [74,75] was used to compare repeated measurements (reaction times across task types) within groups.

A general analysis of the data irrespective of task types and motion directions revealed that **location-based vibrations** were more effective and efficient in alerting participants to moving objects. The *detection rate* was 71% (versus 46.3%), and the average *DT* was 2.5 s shorter compared to the **pattern-based vibration** condition (Table 2). *RT* was also shorter, with an average reduction of 1.2 s, the difference supported by Mann–Whitney U-test statistic (p<0.01, Cliff’s delta = 0.58 showing a large effect size).

### 6.1. Motion Directions

**Figure 7:**
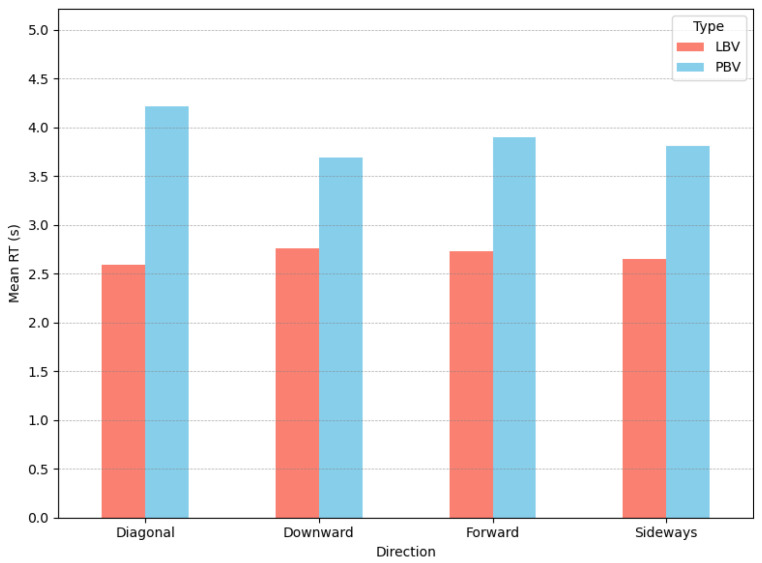
Mean reaction times per group across directions.

**Figure 8:**
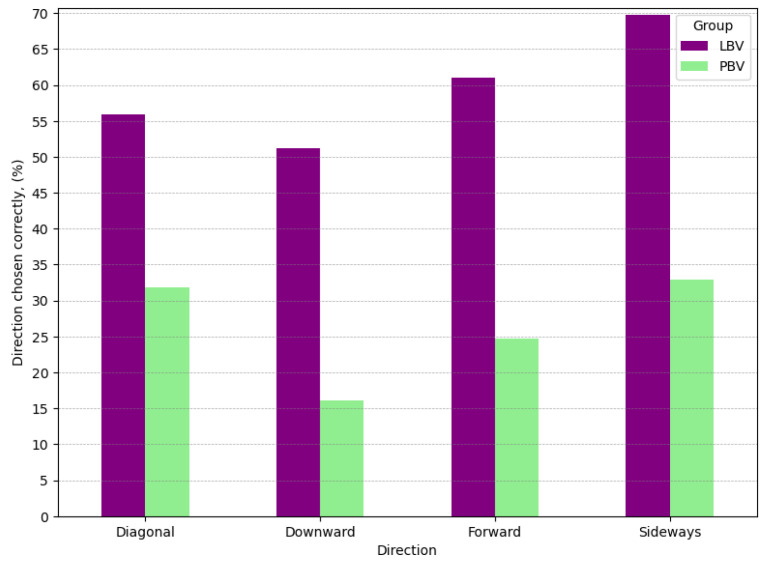
Rates of correctly chosen direction per group across directions.

Analysing the data from the perspective of motion direction reveals several noteworthy trends. Firstly, during each task across all motion directions, the LBV group reacted faster with effect sizes varying from medium for *downward* direction to large in *diagonal* directions (Table 3). Secondly, in the **PBV** group, *RT* varied significantly across directions (Table 4, Figure 7). Within the group, the *downward* pattern was particularly efficient, with RT more than 500 ms faster on average than others. In contrast, *RT* in the **LBV** group were more uniform, the largest difference between directions being ∼160 ms. Coupled with *detection rate* median *RT* further suggest that *forward* motion pattern was the most efficient (Figure 8).

Both groups exhibited variability in *DT*. *Downward* motion, despite being the fastest to respond to, had the lowest *detection rate*, with only every fifth object spotted. This pattern aligns with the low *accuracy rates* for initial directional responses, where participants often failed to look or turn in the correct direction after receiving an alert. In the **LBV** group, the *detection rates* were significantly higher across all directions compared to the **PBV** group. *Diagonal* directions were the most successfully detected, while *sideways* directions had the lowest detection rate.

Lastly, the *correctness of directional responses* leaves room for improvement in both groups. The **LBV** group demonstrated a clear advantage, with participants twice as likely to choose the correct direction compared to the **PBV** group.

### 6.2. Task Types and Urgency

We analysed reaction times across different types of tasks. It was hypothesised that the **memorisation and recall** task, potentially being the most cognitively demanding, would result in increased *RT*. The task type had a statistically significant influence only in **PBV** group, showing slightly slower reactions during Task 3 compared to Task 1 or 2 (Table 5).

Regardless of urgency and direction, groups show a similar tendency in the correctly chosen direction percentage across the tasks. Thus, in the **PBV** group, the percentages were 27.12%, 23.01%, and 28.57% per Task 1, 2 and, 3 respectively, while in the **LBV** group, the percentages were 57.27%, 54.13%, and 66.06%. Interestingly, participants chose direction correctly most often during the memorisation task and least often during the mathematical task.

In terms of urgency, ART ANOVA for within-group RT comparison revealed that regardless of motion direction, the high and low urgency *RT* in the **PBV** group were different (est.=−32.3, SE=10.2, p=0.01), as well as medium and low (est.=−24.8, SE=10.1, p=0.04), showing slower reaction to lower urgency. When comparing *RT* within directions, only *diagonal* motion in the **PBV** group showed differences between high and low (est.=−12.7, SE=5.4, p=0.05), and medium and low (est.=−12.6, SE=5.3, p=0.05) urgency levels. No significant difference was found in the LBV group.

Percentage of correctly chosen directions distributed differently across urgency levels in both groups. Thus, in the **PBV** group, percentages were 22.52%, 28.81%, and 26.55% for high, medium and low urgency levels, respectively, while in the **LBV** group, percentages were 62.39%, 59.81%, and 55.36%, showing a decrease in right decisions with the signal slowing.

## 7. Discussion

The first experiment provided data necessary to construct haptic motion patterns for comparison with simple vibrations. Since no specific pattern or feature set emerged as clearly superior, we selected the pattern preferred by a marginal majority of the participants. We also observed a negative correlation of perceived urgency with BD, which supported our decision to manipulate BD values to represent three levels of urgency and to keep the vibration frequency constant in the second experiment.

### 7.1. Effect of the Haptic Signal Type

The results indicate that location-based haptic alerts were more effective in terms of detection rates and more efficient in terms of reaction times. The percentage of correctly chosen reaction directions suggests that location-based vibrations were clearer for participants. It was also observed that patterns required more concentration to mentally track the direction of the vibration “moving” across the skin. This may be due to a larger population of sensory receptors involved in the sequential delivery of motion-associated information [76]. The result is in line with the research by Faugloire et al. [77] who, on the abdomen example, showed that apparent movement representing one straight line is associated with a decrease in recognition time and accuracy, which in turn could cause a more complex tactile remapping process [78,79]—combining tactile information with proprioception.

The *forward* direction pattern, different from the rest because of its non-linear design, nevertheless showed interesting results within its group. It outperformed simpler, less ambiguous *downward* and *sideways* patterns in *detection rate* and earned 100% attention of all participants unlike any other stimulus. This may be due to *downward* and *sideways* patterns being more monotonous in terms of actuator group activation. Monotony has been associated with neuronal habituation and adaptation, which are characterised by a weaker neuronal response compared to more diverse stimuli in several modalities, including somatosensory modality [80].

Our data suggest that stimulation of specific upper back locations is more effective in describing directions. Still, the percentage of correctly chosen directions while reacting (ranging from 51% to 69%) does not prove this method being a highly intuitive way of delivering information during task execution. Furthermore, participants in the LVB group reported occasional confusion between the upper middle actuator (*downward* motion) and the central actuator (*forward* motion) when receiving one of the two stimuli. Discerning between upper and middle locations when only one is stimulated does not prove unequivocal. This issue could be addressed by repositioning the upper actuator, perhaps on the neck or head, to reduce the ambiguity caused by the proximity of these two actuators.

### 7.2. Effect of the Task Type

Task type influenced reaction times in the PBV group but not in the LBV group. Since both groups performed the same tasks, this suggests that interpreting patterned haptic signals required more cognitive resources than processing location-based signals, a conclusion supported by Kahneman’s theory of attention [81].

### 7.3. Effect of Urgency

In terms of urgency, only a marginal statistically significant difference was observed in the PBV group and estimated 24 and 32 ms between *low and high* and *medium and low* urgency levels correspondingly. However, further analysis showed that only diagonal motion’s RT varied across urgency levels. Presumably, this can be attributed to an increased number of actuator groups and, considering the funnelling effect [47], less obvious trajectory of movement. The location-based vibrations all seemed to cause similar reactions.

It is possible that the instructions (“react to the alert when you feel it”) reduced the effect of urgency, with participants responding as soon as they sensed any cue. This could explain the low percentage of correctly chosen directions in the PBV group. In real-life scenarios, it is likely that people would also react to a hazard alert as quickly as possible. Therefore, information delivered within first seconds is bound to be unambiguous to eliminate possibilities of missing a hazard because of the wrong choice of direction.

Lastly, while the difference in the number of “skipped” reactions between the groups was small, they are worth considering. It may be that shorter alerts are more easily ignored, leading to this difference.

### 7.4. Broader Applications

Although our experiments focused on construction-related scenarios, the results may extend to other safety-critical domains. In mining, where noise and limited visibility often reduce the reliability of auditory or visual signals, haptic alerts could help workers notice approaching machinery or hazardous gas leaks. Manufacturing environments may similarly benefit, for instance, when operators need timely warnings about malfunctioning equipment or unexpected movement of robotic arms while their attention is visually occupied. In transportation, location-based cues could provide additional support by drawing attention to blind spots or potential collisions, particularly in situations of high cognitive demand. These examples suggest that haptic alerts can complement existing channels, although their practical value will depend on careful integration into the sensory and cognitive load of each work setting.

### 7.5. Limitations

Several factors define the scope of our findings. As an exploratory laboratory study, we recruited a limited number of university students whose sensory habits and risk perception differ from those of professional construction workers. While this limits direct generalisability, it was an appropriate first step of systematic investigation of haptic signal design under controlled conditions.

To maintain experimental control and prevent fatigue or sensory desensitisation, we restricted the number of signal features and repetitions. Minor variations in pattern repeat times (two versus three repetitions) balanced task length and engagement, without clear evidence of bias.

Although an immersive virtual environment enhanced realism, participants remained stationary and tactile feedback was limited to a 9-actuator vest without interference from tool or machinery simulations. The task complexity was simplified but sufficient to demonstrate the cognitive involvement effects on reaction times.

Within this defined scope, our results demonstrate that location-based vibration signals—falling within the framework of hermeneutic mediation haptics—can serve as effective and efficient safety alerts for novice users, even when interpretation is required.

### 7.6. Future Work

Future research could extend this work by validating haptic alerting systems with professional workers in real-world or highly realistic environments, enabling direct assessment of their effectiveness and usability in the target population. A number of factors inherent to work sites such as physical activity, vibrations produced by hand tools or machinery could possibly reveal benefits of pattern-based vibration alerts, less monotonous comparing to counterparts. Building on the initial findings, future studies can also design tasks that more closely replicate the cognitive and physical demands of construction or other types of work, supporting deeper insights into task-specific haptic performance. Investigating haptic signal perception during user movement, potentially through alternative vest designs or improved actuator attachment methods, offers another important avenue to ensure robustness under dynamic conditions. Furthermore, further exploration of how different haptic mediation types interact with varying cognitive loads or how much of mental resources they require could inform the development of adaptive, context-aware alerting systems. Finally, future research should consider practical concerns about deploying a haptic vest on workers such as variety of clothing and its effects on vibration sensing and test different vest configurations.

## 8. Conclusions

This research advances haptic safety alerting through the following contributions:

- Urgency Modulation: We found a significant negative correlation between perceived urgency burst duration, which suggests a recommendation of using shorter burst durations to encode higher urgency.
- Comparison of Alerting Strategies: We conducted a novel comparison between pattern-based and location-based haptic alerts in a simulated high-risk work environment.
- Location-Based Alerts Superiority: Our results suggest location-based alerts outperform motion patterns in conveying directional hazard information, particularly for novice users.
- Pattern Design Insights: Our experiment provided initial insights into how motion patterns can be constructed to simulate directional movement on the back.
- Implications for Wearable Safety Systems: The findings inform the design of wearable haptic safety systems, offering evidence-based trade-offs and guidance for high-risk environments.

These findings contribute to a growing body of research advocating haptic feedback as a crucial modality to enhance safety and situation awareness in demanding work environments which include construction, manufacturing, and mining domains. By systematically investigating and comparing different haptic alerting strategies, this work paves the way for the development of more effective, intuitive, and user-centred wearable safety technologies, ultimately contributing to a reduction in-workplace accidents and improved worker well-being.

## Figures and Tables

**Table 1:** PERMANOVA statistics for patterns across different sets of features.

Set of Features	Pattern b (Downward)	Patterns c–d (Sideways)	Patterns e–f (Diagonal)
Frequency + BD + IBI	pseudo-F = 0.27, *p* = 0.6	pseudo-F = 0.65, *p* = 0.4	pseudo-F = 0.01, *p* = 0.9
Frequency + BD	pseudo-F = 0.27, *p* = 0.6	pseudo-F = 0.65, *p* = 0.4	pseudo-F = 0.01, *p* = 0.9
BD + IBI	pseudo-F = 0.73, *p* = 0.4	pseudo-F = 0.10, *p* = 0.8	**pseudo-F = 21.07, ** ***p*** **< 0.01**

**Table 2:** Comparison of detection and reaction measures.

	PBV	LBV
**Mean DT**, s	6.659	4.163
**Median DT**, s	6	4
**SD**, s	2.69	1.13
**IQR**, s	2	2
**Mean RT**, s	3.907	2.684
**Median RT**, s	4	3
**SD**, s	1.41	0.73
**IQR**, s	1	1
**Detection rate**, %	46.33	71.10
**Reactions**, %	97.18	95.47

**Table 3:** Comparison of reaction times between PBV and LBV across tasks and motion directions.

Task	Test	Downward	Sideways	Diagonal	Forward
**Task 1**	U/δ	579.0, p<0.01 (δ = 0.37)	576.0, p<0.01 (δ = 0.52)	745.0, p<0.01 (δ = 0.77)	622.5, p<0.01 (δ = 0.43)
**Task 2**	U/δ	644.5, p<0.01 (δ = 0.58)	483.5, p<0.01 (δ = 0.48)	713.0, p<0.01 (δ = 0.75)	596.5, p<0.01 (δ = 0.47)
**Task 3**	U/δ	671.0, p<0.01 (δ = 0.59)	552.0, p<0.01 (δ = 0.76)	698.5, p<0.01 (δ = 0.72)	601.5, p<0.01 (δ = 0.54)

U—Mann–Whitney U-test value, δ—Cliff’s delta (positive values indicate longer RTs in PBV)

**Table 4:** Direction-wise statistic: detection and reaction rates.

	Pattern-Based Vibration				Location-Based Vibration			
	**Downward**	**Sideways**	**Diagonal**	**Forward**	**Downward**	**Sideways**	**Diagonal**	**Forward**
**Mean DT**, s	**4.684**	5.412	7.483	7.264	3.896	**3.614**	4.707	4.156
**Median DT**, s	5.000	5.000	7.000	7.000	4.000	4.000	4.000	4.000
**SD**, s	0.70	2.07	3.18	2.81	0.89	0.92	1.17	1.13
**IQR**, s	0.5	2.0	2.0	2.0	1.0	1.0	2.0	2.0
**Mean RT**, s	**3.690**	3.810	4.216	3.900	2.756	2.649	**2.595**	2.732
**Median RT**, s	4.000	4.000	4.000	3.500	3.000	3.000	3.000	3.000
**SD**, s	1.46	1.38	1.42	1.42	0.69	0.70	0.69	0.72
**IQR**, s	2.0	1.0	2.0	1.75	1.0	1.0	1.0	1.0
**Detection rate**, %	21.35	39.53	**65.17**	58.89	53.33	64.77	**94.25**	70.45
**Reactions**, %	93.26	91.86	98.88	**100**	97.78	89.77	**98.85**	95.45
**Direction chosen correctly**, %	16.09	**32.91**	31.82	24.72	51.16	**69.74**	55.95	60.98

**Table 5:** ART-ANOVA results of reaction time comparison across tasks within PBV and LBV groups.

	PBV			LBV		
	**Estimate**	**SE**	***p***	**Estimate**	**SE**	***p***
**Task 1–Task 2**	−20.3	9.7	0.09	−5.5	8.8	0.8
**Task 1–Task 3**	−54.7	9.8	**<0.01**	−12.3	8.8	0.3
**Task 2–Task 3**	−34.4	9.9	**<0.01**	−6.8	8.8	0.7

## Data Availability

The datasets presented in this article are not readily available because due to technical and time limitations. Requests to access the datasets should be directed to Albina Rurenko (albina.rurenko@tuni.fi).

## Abbreviations

The following abbreviations are used in this manuscript:

BD	Burst duration
IBI	Inter-burst interval
JND	Just noticeable difference
ANOVA	Analysis of variance
PERMANOVA	Permutational multivariate ANOVA
PBV	Pattern-based vibration
LBV	Location-based vibration
VR	Virtual reality
IPI	Inter-pulse interval
RT	Reaction time
DT	Detection time
ART-ANOVA	Aligned rank transform ANOVA
